# *Ustilago maydis* populations tracked maize through domestication and cultivation in the Americas

**DOI:** 10.1098/rspb.2007.1636

**Published:** 2008-02-05

**Authors:** Andrew B Munkacsi, Sam Stoxen, Georgiana May

**Affiliations:** 1 Plant Biological Sciences Graduate Program, University of MinnesotaSt Paul, MN 55108, USA; 2 The Center for Community Genetics, University of MinnesotaSt Paul, MN 55108, USA; 3 Department of Ecology, Evolution and Behavior, University of MinnesotaSt Paul, MN 55108, USA

**Keywords:** host and pathogen coevolution, crop domestication, smut fungi, agriculture, host expansion, ybp, years before present, mya, million years ago, SSR, simple sequence repeat, SMM, stepwise mutation model

## Abstract

The domestication of crops and the development of agricultural societies not only brought about major changes in human interactions with the environment but also in plants' interactions with the diseases that challenge them. We evaluated the impact of the domestication of maize from teosinte and the widespread cultivation of maize on the historical demography of *Ustilago maydis*, a fungal pathogen of maize. To determine the evolutionary response of the pathogen's populations, we obtained multilocus genotypes for 1088 *U. maydis* diploid individuals from two teosinte subspecies in Mexico and from maize in Mexico and throughout the Americas. Results identified five major *U. maydis* populations: two in Mexico; two in South America; and one in the United States. The two populations in Mexico diverged from the other populations at times comparable to those for the domestication of maize at 6000–10 000 years before present. Maize domestication and agriculture enforced sweeping changes in *U. maydis* populations such that the standing variation in extant pathogen populations reflects evolution only since the time of the crop's domestication.

## 1. Introduction

The domestication of plants and animals and the subsequent development of agriculture brought major changes in human societies (Smith 1995), their relationship to the natural environment and the evolution of disease. For example, the development of slash and burn agriculture in central Africa has been implicated in the evolution of more virulent forms of the malarial parasite, speciation in *Anopheles* mosquitoes and subsequent evolution of some forms of malarial resistance in humans (Tishkoff *et al*. 2001). As nascent as are archaeological studies of human disease, much less is known of agriculture's impact on plant disease dynamics. Two recent reports date speciation in plant pathogenic fungi to the times and locations of their host crop's domestication (Couch *et al*. 2005; Stukenbrock *et al*. 2007), demonstrating that the crop domestication has had tremendous impacts on the pathogen evolution over relatively short periods of time. We examined the impacts of maize domestication and subsequent cultivation throughout the Americas on the evolution of the corn smut fungus *Ustilago maydis*, an obligate fungal pathogen of maize.

Previously, we dated the divergence of *U. maydis* from its sister taxon, *Ustilogo bouriquetii*, at 10–25 million years ago (mya; Munkacsi *et al*. 2007); thus, *U. maydis* must have originated long before the time of maize domestication only 6000–10 000 years before present (ybp; reviewed in Staller *et al*. 2006). *Ustilago maydis* was probably a pathogen on the teosinte progenitor of maize, *Zea mays* ssp. *parviglumis*, because most *Ustilago* species and the members of related genera are host-specific pathogens on grasses of the Poaceae (Stoll *et al*. 2005), and the current host range of *U. maydis* is limited to *Z. mays* subspecies (Duran 1987; this report). We asked whether the evidence of this ancient coevolutionary history is apparent in contemporary populations or alternatively, if the dynamic response of pathogen populations to modern crop plant management has written over the historical patterns of variation as it apparently has in other systems (e.g. Roelfs & Groth 1980; Zhu *et al*. 2000; Kolmer *et al*. 2004; Stukenbrock *et al*. 2006). Contemporary *U. maydis* populations demonstrate little long-distance migration (Voth *et al*. 2006), yet maintain substantial variation and exhibit low levels of local inbreeding (Barnes *et al*. 2004), suggesting that modern practices have not homogenized corn smut populations as they have for other crop pathogens (e.g. Stukenbrock *et al*. 2006). The uneven impact of domestication and early agriculture across pathosystems is further suggested by the findings that the geographical origins and centre of diversity correspond for some pathogens (Banke & McDonald 2005; Stukenbrock *et al*. 2007), but not for others (Zaffarano *et al*. 2006; Brunner *et al*. 2007). The extent to which a pathogen's demographic history might constrain or amplify the responses to demographic and genetic changes in plant host populations is not well understood.

Domestication transformed the teosinte *Z. m*. ssp. *parviglumis* into maize (*Z. m*. ssp. *mays*) in the Balsas River valley of Mexico (Matsuoka *et al*. 2002; Doebley *et al*. 2006). As maize was subsequently moved to South America 4000–7000 ybp (Piperno & Pearsall 1998; Staller *et al*. 2006) and to the United States 1000–3000 ybp (Staller *et al*. 2006), the crop was adapted to new environments and uses. In the early part of the twentieth century, strong efforts for resistance breeding in maize to *U. maydis* incorporated a number of quantitative loci from a variety of genetic backgrounds (Lubberstedt *et al*. 1998; Baumgarten 2004; Baumgarten *et al*. 2007). With the native Americans' use of corn smut as a food source (Wilson 1987; Valverde *et al*. 1995; Iltis 2000), varying ecological conditions throughout the Americas and the quantitative nature of *U. maydis* resistance in maize (Lubberstedt *et al*. 1998; Baumgarten *et al*. 2007), selection on *U. maydis* populations has been variable over time and geography. Nonetheless, the well-documented history of maize agriculture in the Americas sets upper limits for times of *U. maydis* population expansion as maize is the only host of *U. maydis* outside of Mexico.

We investigate the null hypothesis that the domestication of maize had little measurable impact on the genetic variation that built up in the ancient coevolutionary dynamic between *U. maydis* and its hosts. Under the null hypothesis, we expected that the centre of genetic diversity in current *U. maydis* populations should be in Mexico as this is the apparent centre of origin for *U. maydis* and its ancestral teosinte hosts, the subspecies of *Z. mays*, ssp. *parviglumis* and ssp. *mexicana* (Sánchez *et al*. 1998). Alternatively, if the domestication of maize led to a strong genetic bottleneck of *U. maydis* populations, we expected that extant populations should not date older than the thousands year old history of maize in Mexico and that the diversity of populations in Mexico should not be greater than that in descendent populations. Considering more recent history, we investigate the hypothesis that modern, intensive maize agriculture practised in South America and the United States caused high rates of migration and successive bottlenecks within descendent *U. maydis* populations. We tested the expectation that these populations will exhibit little population substructure and lower levels of genetic diversity than that in Mexico. Pursuant to these hypotheses, we sampled *U. maydis* populations on maize*, Z. m*. ssp. *parviglumis* and *Z. m*. ssp. *mexicana* in Mexico and on maize in Mexico and throughout the Americas, and analysed population genetic structure using 10 simple sequence repeat (SSR) markers. We find little evidence of the ancient evolutionary history of *U. maydis* in Mexico, yet despite the intensive culture of modern maize, the thousands year old imprint of early maize agriculture remains evident in the genetic structure of *U. maydis* populations today.

## 2. Material and methods

### (a) *Ustilago maydis* and sampling

After mating of compatible haploid cells, *U. maydis* forms an infectious dikaryotic hypha and later develops a hypertrophy, or gall, containing diploid teliospores at the site of the localized infection. Our extensive field observations (Baumgarten *et al*. 2007) indicate a single generation per year in regions of annual maize cultivation with very low plant-to-plant spread within a year. Sampling was conducted between 1998 and 2002; 44 field locations were chosen to represent the geographical range of maize domestication and early cultivation, as well as variation in maize landraces and teosinte subspecies, *Z. m*. ssp. *parviglumis* and *Z. m*. ssp. *mexicana*. We sampled one gall (one diploid) per plant directly into 2 ml cryovials in the field, and sampled approximately 25 galls per field for a total of 1088 *U. maydis* individual galls (figure 1*a*; electronic supplementary material, table 1).

### (b) Scoring SSR loci

Ten SSR loci on nine different chromosomes were selected to represent a range of repeat motif lengths and varying levels of diversity appropriate to the possible time scales of this study (Munkacsi *et al*. 2006). The repeat motif characteristics and forward and reverse primer sequences for each locus are provided (electronic supplementary material, table 2). Total genomic DNA was extracted from approximately 10 mg of teliospores. PCR cycle conditions used to amplify fragments from 10 ng of genomic DNA using fluorescent-labelled primers and automated sequencer analysis of PCR fragment sizes (Applied Biosystems model 3100) were the same as described previously (Munkacsi *et al*. 2006). Fragment (allele) size ±0.5 bp was determined with Genescan Analysis software (v. 3.7, Applied Biosystems, Inc.). Background amplicons were distinguished as chromatogram peaks not larger than 25% of the tallest peak for each locus and amplifications were rerun if more than two peaks per diploid genotype were observed.

### (c) Identification of populations

Genetically distinct populations were identified using a Bayesian clustering method implemented in Structure v. 2.0 (Pritchard *et al*. 2000). For each *K* number of populations modelled, individual genotypes are assigned membership in a population as a function of allele frequencies and the proportion of an individual's genotype drawn from each of the *K* populations. Individuals are assigned to clusters to maximize Hardy–Weinberg equilibrium, linkage equilibrium and genetic homogeneity within each cluster. For each parameter value of *K*, admixture and correlation between alleles were varied in triplicate runs of 100 000 iterations with a burn-in period of 30 000 iterations.

### (d) Marker and population genetic analyses

We compared the observed heterozygosity and the number of alleles for each population to expectations under a neutral equilibrium model, analogous to the Ewens–Watterson test for DNA sequence variation (Ewens 1972; Watterson 1978). A deficiency or excess of heterozygosity relative to the number of alleles was evaluated against neutral expectations and a 95% confidence interval generated by permutation.

We used Arlequin (Schneider *et al*. 2000) to estimate the following: genetic diversity as the unbiased mean expected heterozygosity (*H*; Nei 1987); number of total alleles; number of rare alleles (frequency <0.05); number of private alleles (those found in only one population); and levels of genetic differentiation (*R*_ST_). The *R*_ST_ statistic uses variation in allele sizes and incorporates the stepwise mutation model (SMM) to evaluate the proportion of the total genetic variation that is found in a subpopulation (Slatkin 1995). The significant difference of *R*_ST_ values from zero, at *p*<0.05, was determined with 1000 permutations of individuals between populations in Arlequin.

We used BayesAss+ (Wilson & Rannala 2003) to evaluate migration rates per generation as the mean proportion of one population entering another population and as the mean proportion of the population staying in that local population. Linkage equilibrium and non-overlapping generations but not Hardy–Weinberg equilibrium are assumed. We ran 3×10^6^ iterations, discarding the first 10^6^ iterations as burn-in, and thereafter samples were collected every 2000 iterations to infer population allele frequencies and migrant proportions. One generation per year was assumed.

We tested for the effect of population bottlenecks using Bottleneck (Cornuet & Luikart 1996) under SMM. The test examines evidence for an observed excess heterozygosity over that expected from allele numbers because with small founding populations, the number of alleles is reduced more than heterozygosity (Nei 1987). Significant deviation in observed heterozygosity from that expected was evaluated with a two-tailed test of Wilcoxon signed-rank test across loci because a deficiency of heterozygosity will occur with long-term inbreeding. We tested for linkage disequilibrium by determining the index of association (*I*_A_) using Multilocus (v. 1.2) with significance evaluated by permutation (Agapow & Burt 2001).

We conducted the *k*- and *g*-test (Reich *et al*. 1999) using KGTESTS (Bilgin 2007) to evaluate evidence for population expansion within each major population. Similar to mismatch distributions for DNA sequences, the *k*-test compares the observed distribution of allele sizes that is unimodal in an expanding population with the expected distribution of allele sizes that is multimodal in a population of constant size. The measure *k*, related to kurtosis, is centred on 0, and the proportion of loci that gives negative values is evaluated with a binomial test (Reich *et al*. 1999). The *g*-test uses the observed variation across loci in variance of allele sizes with the rationale that variation across loci will be greater in a population of constant size than in a newly expanding population (Reich *et al*. 1999). The *g* value is calculated as a ratio of the observed variation across loci to that expected under populations of constant size and is evaluated against a table of 5th percentile cut-off values (*p*<0.05) obtained by simulation for different sample sizes and numbers of loci and is robust to variation in population size (table 1 in Reich *et al*. 1999).

### (e) Population divergence dates

We adopted the method of Zhivotovsky (2001) to obtain earlier and later bounds for divergence time between each pairwise combination of populations. Times of divergence (*T*_D_) are estimated as: $T_{\text{D}}=({\bar{D}}_{1}/2\bar{w})-({\bar{V}}_{0}/\bar{w})$, where *T*_D_ is the estimate of divergence time in generations, ${\bar{D}}_{1}$ is the average squared difference in allele size between pairs of sampled alleles over all the loci (Goldstein *et al*. 1995) and ${\bar{V}}_{0}$ is the estimate of average variation in repeat number across loci in the ancestral population. The earlier date is obtained under the assumption that *V*_0_=0 and the more recent date is obtained under the assumption that *V*_0_ is the same as allelic variation among extant populations. The estimates are not affected by population growth but are affected by gene flow. Using these extremes as estimates for *V*_0_, the resulting bounds can be considered conservative estimates of divergence time. We estimated the average mutation rate over SSR loci $\left(\bar{w}\right)$ by two methods, both assuming one generation per year. The regression for the number of repeats in an SSR locus and rate of evolution in eqn 1 of Thuillet *et al*. (2005) generates an estimate of rate without assuming a calibration date. A second estimate of rate was obtained using the variance in allele sizes as an estimate of 2 *Nμ* for USA populations (Valdes *et al*. 1993) and a calibration time of 2000 years for *U. maydis* populations in the United States.

## 3. Results

We obtained smut gall samples from three *Z. m*. ssp. *parviglumis* populations and three *Z. m*. ssp. *mexicana* populations in Mexico (figure 1). The *Z. m*. ssp. *mexicana* plants occurred as weeds in maize fields and galls were also collected from those maize plants. *Ustilago maydis* infections on teosinte plants are characterized by one to very few small galls on seeds and low infection rates (approx. 0.1% of plants). Infections on maize plants are characterized by numerous, larger galls occurring on any plant organ and at higher infection rates (approx. 3% of plants). Obtaining DNA from small gall samples representing single infection events, we evaluated a total of 1088 diploid teliospore collections (electronic supplementary material, table 1) from all plant populations for variation at 10 SSR loci (electronic supplementary material, table 2).

### (a) Structure identifies five major populations

Structure assigns diploid individuals membership to genetically distinct clusters (populations) without reference to the geographical location of the collections (Pritchard *et al*. 2000). Modelling *K*=1–10 populations, we found the best support for *K*=5 genetically distinct clusters or ‘major populations’ (figure 1*b*) based on the recommended criteria that the rate of change in the log probability of the data between successive *K* values (Δ*K*; Evanno *et al*. 2005) was the greatest in the interval of four to five populations, and thus that it began to plateau (Pritchard *et al*. 2000) at *K*=5. Varying the degree of admixture between populations from 0.05 to 0.50 yielded little effect on the estimated log probability or Δ*K* values. Three independent runs for each *K* yielded consistent results.

The five major populations identified in Structure represent large geographical regions of Mexico, South America and the United States (figure 1*a*). In Mexico, we found evidence for two quite distinct populations, AG-T and MEX. The AG-T population was limited to collections from one *Z. m*. ssp. *parviglumis* population near Amates Grandes, Guerrero (*n*=30). The MEX population comprised all collections from the remaining 14 fields in Mexico (*n*=324) and included nine fields of Mexican maize landraces and five fields from the two teosinte taxa. In South America, we identified two major populations, SA1 and SA2, collected from South American maize landraces. The SA1 population comprised collections from seven maize fields (*n*=218) and the SA2 population comprised collections from six maize fields (*n*=154). SA1 and SA2 are genetically but not geographically distinct from each other (figure 1*b*). The fifth major population, USA, comprised collections from elite maize hybrid lines in 16 maize fields in the United States (*n*=362).

Using Arlequin (Schneider *et al*. 2000), we identified between 1 and 40 alleles and estimated expected heterozygosity (*H*) values between 0.04 and 0.91 per major population, across the five major populations and 10 SSR loci. Results of the Ewens–Watterson test (Ewens 1972; Watterson 1978) showed that observed heterozygosity and number of alleles fit neutral expectations within the 95% CI for all loci except locus 1.64 in the AG-T population that demonstrated a slight excess heterozygosity (electronic supplementary material, figure 1).

To quantitatively compare the levels of genetic differentiation between the five major populations, we calculated *R*_ST_ values across all 10 SSR loci using Arlequin and assuming SMM (Schneider *et al*. 2000). The *R*_ST_ evaluates the distribution of SSR variation within and between populations using allele sizes and assumes random mating (Slatkin 1995). High levels of genetic differentiation (*R*_ST_>0.15) were found among all comparisons except that of MEX and SA1 (*R*_ST_=0.07) and of SA1 and SA2 (*R*_ST_=0.11); all were significantly greater than zero. The AG-T population was the most strongly differentiated one from all other populations (mean pairwise *R*_ST_=0.53±0.07). Interestingly, both Structure and *R*_ST_ results show that the USA population is more strongly differentiated from the MEX population than are either SA1 or SA2.

To determine whether unique field populations might strongly affect the *R*_ST_ and Structure results for major populations, we examined *R*_ST_ values by field. Consistently low *R*_ST_ levels were found within USA (mean pairwise *R*_ST_=0.01±0.02), and low to moderate *R*_ST_ levels were found within SA1 and MEX (mean pairwise *R*_ST_=0.08±0.10 and 0.08±0.03, respectively); we concluded that local fields did not strongly affect those results. However, we did obtain higher *R*_ST_ levels for fields within SA2 (mean pairwise *R*_ST_=0.21±0.14), motivating the investigation of substructure described below. Together, Structure and *R*_ST_ results demonstrate a remarkable degree of geographical structure in *U. maydis* populations given the very short time frame that maize has been cultivated outside of Mexico.

### (b) South American populations demonstrate substructure

We next used Structure to analyse SSR variation within regions. We found little support for more than one genetic cluster (subpopulation) within the USA, MEX or AG-T populations. In contrast, analysing data for all South American collections together, *K*=6 subpopulations were supported and broke up SA1 and SA2 each into three subpopulations (figure 1*c*). SA1 consists of subpopulations 1 (U-T, U-LE, A-T), 2 (P-H, E-Q, P-U) and 3 (B-C, P-S), and SA2 consists of subpopulations 4 (B-SC, B-SL, B-I), 5 (B-P) and 6 (P-CH). No SA1 field population was grouped with SA2 field populations except E-Q, which shows evidence of mixed ancestry (figure 1*b*). Interestingly, substructure by landrace is not apparent while three subpopulations (1, 4 and 6) consisted of *U. maydis* collections from maize growing below 800 m and three subpopulations (2, 3 and 5) consisted of collections from maize growing in the Andes Mountains above 2300 m (figure 1*c*; see table 1 in the electronic supplementary material for landrace and elevation data).

### (c) Genetic diversity is not greater in Mexico

We assumed that Mexico is the centre of origin for *U. maydis* and asked whether the genetic diversity of *U. maydis* populations is greater in Mexico than in descendent populations of South America and the United States. Across the ten SSR loci, the MEX population had approximately the same expected heterozygosity (*H*; Nei 1987) as the descendent USA, SA1 and SA2 populations. The AG-T population demonstrated significantly lower expected heterozygosity than any other population (table 1). The greatest numbers of total, rare and private alleles were found in the USA population (table 1), largely due to the extraordinary levels of variation at two loci (1.89, 1.206; electronic supplementary material, table 2) with 31 and 40 alleles each. Even without data for these two loci included, levels of variation observed in the USA, SA1 or SA2 populations are the same as in MEX and higher than that in AG-T. Interestingly, five loci were fixed for one allele in the AG-T population and there were no rare or private alleles, suggesting an isolated and inbreeding population.

### (d) Host subspecies contribute to genetic structure of U. maydis populations in Mexico

To evaluate the contribution of host variation to the genetic structure of *U. maydis* populations in Mexico, we estimated *R*_ST_ values among collections from maize (*n*=234), *Z. m*. ssp. *mexicana* (*n*=66) and *Z. m*. ssp. *parviglumis* (*n*=54). Comparing all maize (*n*=234) and all teosinte collections (*n*=120), we obtained significant, moderate levels of genetic differentiation (*R*_ST_=0.14). Because the AG-T population is so distinct, we analysed all maize-infecting collections (*n*=234) versus all teosinte-infecting collections without the AG-T samples (*n*=90) and obtained a lower but still significant level of differentiation (*R*_ST_=0.10). Separate comparisons of *U. maydis* populations on maize with those on *Z. m*. ssp. *mexicana* or *Z. m*. ssp. *parviglumis* (without AG-T) yielded similar and significant values (*R*_ST_=0.10 and 0.08, respectively). These results show low to moderate levels of differentiation of *U. maydis* populations by host subspecies, perhaps surprising, since *U. maydis* collections were obtained from both *Z. m*. ssp. *mexicana* and maize growing in the same fields, and since *Z. m*. ssp. *parviglumis* is the direct ancestor of maize.

### (e) Little migration occurs between major populations

Cognizant of continuing trade of maize in the Americas and the potential for long-distance wind-borne smut spore dispersal, we used BayesAss+ (Wilson & Rannala 2003) to estimate bidirectional migration rates among the five major populations. Results show little evidence of ongoing gene flow among the major populations because each retains a mean of 99% of its own population per generation, with the exception of SA1. SA1 retains approximately 95% of its own population and acquires approximately 4% of the SA2 population per generation (table 2). These results suggest that neither wind-borne spores nor contemporary agricultural trade have lead to significant levels of long-distance migration between major *U. maydis* populations.

### (f) Historical demographic processes affected extant population structure

Bottleneck uses a Bayesian framework to evaluate the evidence for founder effects in descendent populations as observed excess heterozygosity over that expected for the number of alleles at each locus. Bottleneck removes data for loci fixed for one allele (Cornuet & Luikart 1996). Evidence for bottlenecks was strong for eight to nine out of nine loci in each of the major populations except AG-T (table 1). In AG-T, five out of the 10 loci were fixed for one allele, and the evidence for bottlenecks was not significant at the remaining five loci.

We evaluated disequilibrium association of alleles across loci using the measure *I*_A_ (Agapow & Burt 2001) and found evidence of significant disequilibrium within all major populations (table 1). However, varied causes underlie apparent disequilibrium. The *I*_A_ statistic is probably significant in the AG-T and MEX populations owing to low heterozygosity, sampling across subpopulations in SA1 and SA2 and insufficient time for random association of alleles in the USA population.

We next used the *k*- and *g*-tests to examine the evidence for population expansion (Reich *et al*. 1999). The *k*-test (variation within loci) returned significant results for population expansion for all five major populations (table 1). The *g* values (variation among loci) were large and not significant for any of the major populations (table 1). As a ratio, the *g*-test lacks power to distinguish from the null hypothesis; however, the non-significant *g*-test results could also result from uneven effects of bottlenecks and expansion across each major population. The bottleneck and expansion *k*-test results give evidence for recent bottlenecks and subsequent expansion in Mexico, and in descendent populations in the United States and South America.

Together, evidence for low migration and strong subdivision between large geographical regions and recent population bottlenecks within those regions suggest that descendent populations of *U. maydis* have been largely isolated since the establishment on their maize host populations.

### (g) Corn smut populations are as old as maize agriculture and not much older

We used two approaches to estimate SSR mutation rates, $\bar{w}$, one using a calibration date and the other based on regression analysis in plants Thuillet *et al*. 2005. With either method, we found that the rates for loci 1.89 and 1.206 in USA populations were 5- to 10-fold higher (1.2×10^−3^ and 9.2×10^−4^ mutations per year, respectively) than that estimated for the remaining eight SSR loci. After removing data for 1.89 and 1.206 and assuming SMM, we obtained $\bar{w}$=1.55×10^−4^ steps per year using regression eqn 1 of Thuillet *et al*. (2005), and $\bar{w}$=1.89×10^−4^ steps per year using SSR variance in USA populations as an estimate of 2 *Nμ* (Valdes *et al*. 1993) and a calibration of 2000 years for the USA population. Both are within the range of SSR rates estimated for other organisms (Estoup *et al*. 2002). We used the latter estimate of SSR rate as it involved results and assumptions from our study system.

We estimated divergence times between major populations using the pairwise distance method of Zhivotovsky (2001). The assumption *V*_0_=0 should generate dates approaching the ancient origin of *U. maydis* (10–25 mya) if ancestral variation is still represented in extant populations. Results show the most recent dates at *ca* 1000–2000 ybp in comparisons that included the descendent populations, USA, SA1 and SA2, while the earliest dates at *ca* 9000–10 000 ybp were obtained in comparisons of AG-T with other populations (table 3). Using the lower rates of SSR evolution (1.55×10^−4^), we obtained using Thuillet *et al*. (2005) gives dates not earlier than 12 000 ybp for comparisons including AG-T. Upper bounds obtained for the MEX–USA divergence range up to 6400 ybp and are earlier than expected from the 1000–3000 ybp archaeological dates for maize in the United States (reviewed in Staller *et al*. 2006). All dates of population divergence are on the order expected for maize domestication in Mexico and cultivation in the Americas and none reflect the ancient origin *U. maydis* millions of years ago.

## 4. Discussion

We show that the domestication of maize in Mexico and later expansion of maize throughout the Americas had a dramatic and lasting impact on the evolution of corn smut populations. Although *U. maydis* interacted with its grass hosts over millions of years (Munkacsi *et al*. 2007), extant *U. maydis* populations date not much older than the time of maize domestication at 6000–10 000 ybp (reviewed in Staller *et al*. 2006). Furthermore, the results for dates of divergence, population structure and historical demographic processes show a history of *U. maydis* populations closely associated with that of maize domestication and early cultivation throughout the Americas.

Our results do not provide support for the null hypothesis that maize domestication had little impact on the genetic structure of *U. maydis* populations. The earliest population divergence times dated to only *ca* 10 000 ybp and were found in comparisons that included the *Z. m*. ssp. *parviglumis*-infecting AG-T population. While such recent dates might be obtained if *U. maydis* moved to the *Z. mays* subspecies from an alternate host soon after domestication, hosts other than the *Z. mays* subspecies are not described for *U. maydis* (Duran 1987). Homoplasy is unlikely to account for the recent dates we obtain. We calculated an SSR evolutionary rate of the order of 10^−4^ and dates we obtained are of the order of 10^3^ years. To obtain dates closer to the origin of the species *U. maydis*, which are of the order of 10^7^ years, the rate of evolution at the SSR loci would have to be approximately 10^−8^ steps per year, much too slow for most SSR loci (Estoup *et al*. 2002). Most importantly, the dates obtained with SSR loci for the USA and MEX populations are similar to those we obtained using sequence for the dsRNA virus in *U. maydis* (Voth *et al*. 2006). We infer that the distinct AG-T population is very likely an older, isolated and inbreeding population. With the recent decline and fragmentation of natural teosinte populations (Sánchez *et al*. 1998; Ruiz *et al*. 2001), other small, unique teosinte-infecting *U. maydis* populations must exist, and like AG-T could support little representation of ancient *U. maydis* variation. The population substructure by host subspecies demonstrated here could either represent adaptation to host differences since the time of domestication or a relict of ancestral variation, yet neither scenario pushes dates for the establishment of extant smut populations in Mexico to a time much older than the domestication.

The impact of maize domestication on *U. maydis* populations is further illustrated by the finding that MEX populations harbour no greater genetic diversity than do more recently founded, descendent USA, SA1 and SA2 populations, and similar to these descendents show evidence of population bottlenecks and recent expansion. Other pathosystems also display little correspondence of a species' geographical origin and diversity, implying that the early agricultural practices may often redistribute pathogen variation (Stukenbrock *et al*. 2006; Zaffarano *et al*. 2006). For example, Mexico is the centre of genetic diversity for *Phytophthora infestans*, late blight of potato (Goodwin 1997; Grunwald & Flier 2005) while genealogical results place the origin in the Andes with potatoes (Gomez-Alpizar *et al*. 2007). The contrast between results for diversity and origin points out that standing genetic diversity is strongly affected by recent demographic events and illustrates the challenge of relating the ancient history of crop pathogens to current coevolutionary dynamics. In the case of *U. maydis*, biological evidence that all extant hosts of *U. maydis* originated in Mexico places the ancient origin of *U. maydis* there (Munkacsi *et al*. 2007) whereas our results for demographic history demonstrate that domestication and early agriculture strongly affected both the overall levels of genetic variation and its geographical distribution.

We hypothesized that the intensity of modern agricultural practices and trade would largely obscure the impact of early maize domestication and cultivation in USA and South America. Nonetheless, against a background of low ongoing gene flow between the major populations, we inferred that the pathogen closely tracked maize as native peoples moved the new crop plant to South America (Piperno & Pearsall 1998; Staller *et al*. 2006) and more recently to the United States (Staller *et al*. 2006). In South America, two major populations, SA1 and SA2, each break up into three subpopulations, yet dates of divergence from MEX were estimated at only 1000–7800 ybp. If the SA1 and SA2 populations correspond to the two separate introductions of maize into South America (Frietas *et al*. 2003), then subpopulations within SA1 and SA2 must have independently adapted to high- and low-elevation regions over a relatively short time. For example, both subpopulations 4 and 5 belong to SA2, but lowland subpopulation 4 stretches from Brazil west to Bolivia and is geographically adjacent to the highland subpopulation 5 in the Bolivian Andes. Strikingly, subpopulations 2 (SA1), 3 (SA1) and 5 (SA2) range down the Andes but originated from within the two major populations. The geographical structure demonstrated in South American *U. maydis* subpopulations mirrors the patchwork nature of maize introductions (Piperno & Pearsall 1998; Frietas *et al*. 2003; Iriarte *et al*. 2004; Staller *et al*. 2006), and the apparent, rapid demographic shifts of *U. maydis* populations strongly parallel the intense selection and diversified use of maize.

In contrast to the quite varied structure of *U. maydis* populations in South America, the USA populations we sampled demonstrate the characteristics of recently founded and expanding populations, similar to other pathogen populations (Carbone & Kohn 2001; Brown & Hovmoller 2002; Banke & McDonald 2005; Stukenbrock *et al*. 2006; Brunner *et al*. 2007). However, our results provide a contrast to the human-mediated long-distance dispersal of fungal pathogens (citations above) and instead evidence for dates of divergence, strong differentiation from the MEX population and the presence of unique alleles suggest that USA populations were established at times coincident with the introduction of maize. At 2200–6400 ybp, the dates of divergence we obtained for USA from MEX were broader than we might have expected, but are similar to those of Voth *et al*. (2006) for the dsRNA of *U. maydis* (up to 3700 ybp). Interestingly, smut co-occurs with maize in archaeological remains from southwestern United States at 1500 ybp (Reinhard 2006) and smut spores co-occur with Iroquoian maize pollen at 500–800 ybp in a Canadian lake's sediments (McAndrews & Turton 2007). Considering that the MEX population also shows evidence of recent bottlenecks, we can also infer that ancestral USA populations were founded from a source more diverse than those in the extant MEX population. Variation at the *b* mating-type locus supports that inference as we readily found 20 *b* types in the United States and South America but not more than 13 in Mexico (G. May 2001, unpublished data). Together, the results for divergence dates and genetic structure provide evidence that extant *U. maydis* populations were founded from relatively few but diverse source populations and represent the historic imprints left behind by the expansion of maize agriculture into the United States.

We show that maize domestication and early cultivation enforced dramatic changes in the structure of *U. maydis* populations but without speciation demonstrated for other pathogens (Couch *et al*. 2005; Stukenbrock *et al*. 2007). What then determines the evolutionary response of pathogens to the human impact on host populations and environment? We propose a model for further investigation that the environmental disruption of emerging agricultural systems alters spatial structure of wild and domesticated plant populations such that acquired pathogens are likely to evolve virulence and specialize on the crop. The times and mechanisms by which crops acquire pathogens vary tremendously: at the centre of crop origin from the crop's progenitor species (Stukenbrock *et al*. 2007; this report) or other wild plants (Couch *et al*. 2005), or as a crop is moved into contact with wild pathogen populations (Zaffarano *et al*. 2006). Rather than the acquisition of pathogens *per se*, the most radical impact of altered spatial structure should be that gene flow between pathogen populations on the wild and crop plants will decrease, and breeding within the domesticated host population will increase (Garrett & Mundt 1999; Zhu *et al*. 2000). Further increases in the area of crop planting will result in expanding pathogen populations on domesticated hosts relative to those on wild hosts. Consequently, unlike crop plants themselves (Thuillet *et al*. 2005; Vigouroux *et al*. 2005), genetic diversity in crop pathogen populations will rebound from bottlenecks and support higher levels of genetic diversity than will the populations in surrounding wild plant populations. In addition to these demographic effects, agricultural practices may reduce resistance in host populations (Cohen 2000; Tishkoff *et al*. 2001) or reduce competition (Cohen 2000) and accelerate the evolution of specialized pathogens. For *U. maydis*, we speculate that historical demographic processes and human cultural practices for both the host (Eyre-Walker *et al*. 1998; Fukunaga *et al*. 2005; Wright *et al*. 2005; Staller *et al*. 2006) and the pathogen (Wilson 1987; Valverde *et al*. 1995; Iltis 2000) produced spatially structured populations by host and geography but that gene flow was sufficient to maintain virulence polymorphism (Thrall & Burdon 2003) and impede speciation of the maize-infecting *U. maydis*. More recent maize breeding in the twentieth century incorporated varied maize genetic backgrounds and numerous resistance loci of quantitative effect against *U. maydis* infection (Lubberstedt *et al*. 1998; Wisser *et al*. 2006; Baumgarten *et al*. 2007) and will have contributed to a relatively slow virulence evolution (McDonald & Linde 2002; Carbone & Kohn 2004). Our results demonstrate an immediate impact of crop domestication on pathogen demography and provide a model for the impact of historical events on the ongoing coevolutionary processes among hosts and pathogens.

## Figures and Tables

**Figure 1:**
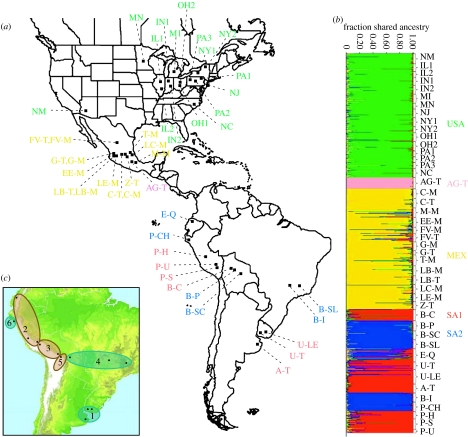
Geographical origin and membership of *U. maydis* individuals in five major populations estimated using Structure (Pritchard *et al*. 2000) (*a*) Collection locations colour-coded according to the membership in five major populations. Collections in Mexico are labelled by location and host; maize (M) or teosinte (T; see table 1 in the electronic supplementary material). (*b*) Estimated membership of each individual to each of the five major populations. (*c*) *Ustilago maydis* subpopulations identified in South America are illustrated by elevation. High-elevation subpopulations (brown circles, more than 2300 m) and low-elevation subpopulations (green circles, less than 800 m) are shown.

**Table 1:** For five major populations, measures of population diversity and genetic structure are given as gene diversity (*H*), numbers of alleles and index of association (*I*_A_) and measures of historic demographic processes are given as bottleneck and expansion test results. (^*^p<0.05; ^**^p<0.01.)

	AG-T	MEX	SA1	SA2	USA
no. individuals	29	275	195	148	341
Nei's *H*^a^	0.17^*^	0.38	0.51	0.42	0.46
no. total alleles	16	59	84	55	123
no. rare alleles	0	31	48	27	70
no. private alleles	0	5	10	1	47
*I*_A_^b^	0.319^*^	−0.436^*^	0.292^*^	0.334^*^	−0.463^*^
bottleneck^c^	3/5 n.s.	8/9^*^	9/9^**^	8/9^*^	9/9^*^
*k*-test^d^	0.008^*^	0.045^*^	0.008^**^	0.008^**^	0.045^*^
*g*-test^d^	n.s.	n.s.	n.s.	n.s.	n.s.

a Unbiased mean expected heterozygosity (Nei 1987); ^*^significant difference in pairwise comparison of AG-T with every other population.

b Index of association (Agapow & Burt 2001).

c Fraction of evaluated loci demonstrating observed heterozygote excess under the SMM; fixed loci were not evaluated.

d *k*- and *g*-tests for expansion (Reich *et al*. 1999).

**Table 2:** Estimates of migration rates between five major populations as the mean of the posterior probabilities of migrants (*m*) per year (95% CI). (Rates of migration from populations listed across the top into populations listed along the side of the table. The proportion of individuals derived from the source population is shown on the diagonal.)

into	from				

	AG-T	MEX	SA1	SA2	USA
AG-T	99 (96.4–100)	0.3 (0–1.6)	0.3 (0–1.7)	0.3 (0–1.6)	0.2 (0–1.7)
MEX	0 (0–0.2)	99 (98.5–100)	0 (0–0.3)	0.3 (0–1.1)	0.2 (0–0.6)
SA1	0 (0–0.4)	0.2 (0–0.9)	95.3 (93.0–97.4)	4.2 (2.2–6.4)	0 (0–0.9)
SA2	0 (0–0.4)	0.1 (0–0.6)	0.3 (0–1.5)	99.2 (97.7–100)	0.3 (0–1.1)
USA	0 (0–0.1)	0 (0–0.2)	0 (0–0.2)	0 (0–0.2)	99.9 (99.6–100)

**Table 3:** Bounds for pairwise divergence times (as ybp) estimated between five major *U. maydis* populations calculated with an average per locus mutation rate of 1.89×10^−4^ steps per year and assuming one generation per year.

	MEX	SA1	SA2	USA
AG-T	5200–9100	5500–12 200	8500–12 000	7500–11 100
MEX		1200–9500	1300–6400	2700–7800
SA1			1300–9100	2800–10 800
SA2				3400–8200
USA				

## Supplementary Material

Electronic supplementary information Sample collection information
