# Supplementary material for: Ustilago maydis populations tracked maize through domestication and cultivation in the Americas
Source: Proc Biol Sci. 2008 Feb 5;275(1638):1037–46. doi: 10.1098/rspb.2007.1636 (PMC2366215; doi:10.1098/rspb.2007.1636)
Supplement: Supplementary file 1 [file rspb20071636s03.doc]

**Supplemental Information - all files**

SI Table 1. Collections of *U. maydis*. The location place name, number of teliospore samples (each from a different plant), host, GPS geographic coordinates, and elevation are provided for each field population.

SI Table 1. continued

SI Table 2. SSR markers. For each marker, the primer sequences, the length of the repeat motif, and chromosomal location are provided.

SI Figure 1. Graphical representation of the Ewens-Watterson neutrality test (Ewens 1972; Watterson 1978). Data points represent observed heterozygosity and total number o alleles per locus per population. The median and 95% confidence interval on observed heterozygosity are shown. One data point outside the confidence interval is for the 1.64 locus in the AG-T population.
